# 自噬对低氧微环境中人肺腺癌A549细胞放疗敏感性的影响

**DOI:** 10.3779/j.issn.1009-3419.2012.11.06

**Published:** 2012-11-20

**Authors:** 勇 李, 丽瑶 徐, 阿桥 蔡, 丽芳 李, 小军 钟

**Affiliations:** 330006 南昌，南昌大学第一附属医院肿瘤科 Department of Oncology, the First Affiliated Hospital of Nanchang University, Nanchang 330006, China

**Keywords:** 肺肿瘤, 自噬, 低氧, 3-甲基腺嘌呤, 放疗敏感性, Lung neoplasms, Autophagy, Hypoxia, 3-Methyladenine (3-MA), Radiation tolerance

## Abstract

**背景与目的:**

已有的研究证明低氧可诱导肿瘤细胞自噬发生，自噬水平与肿瘤放疗敏感性相关，因而调控自噬信号通路是增强放疗敏感性极具潜力的治疗策略。本研究旨在探讨联合应用自噬抑制剂3-甲基腺嘌呤（3-methyladenine, 3-MA）下调低氧环境中人肺腺癌A549细胞的自噬水平后对其放疗敏感性的影响。

**方法:**

实验设低氧对照组及低氧+3-MA（自噬抑制剂）组，分别采用电镜检测自噬体变化，Western blot检测自噬标记蛋白-微管相关蛋白1轻链3（microtubule-associated protein 1 light chain 3, LC3）蛋白表达，分析LC3Ⅱ/LC3Ⅰ比值变化。随后每组再给予直线加速器（0 Gy、2 Gy、4 Gy、6 Gy、8 Gy、10 Gy）照射后采用MTT法检测细胞增殖活性。

**结果:**

与低氧对照组相比，低氧+3-MA组自噬体数量、LC3Ⅱ/LC3Ⅰ比值均下降。给予照射后，与低氧对照组比较，低氧+3-MA组细胞增殖活性下降。

**结论:**

在低氧环境中，A549细胞保护性自噬增加，抑制自噬可增强A549细胞的放疗敏感性。

## Abstract (en)

**Background and objective:**

It has been proven that cancer cell autophagy can be induced by hypoxia. In addition, autophagy was correlated with radiosensitivity. Thus, regulating the autophagy signaling pathway is a potential treatment strategy. The aim of this study is to investigate the effects of combined autophagy inhibitor 3-methyladenine (3-MA) on the radiosensitivity of human adenocarcinoma A549 cells in hypoxia condition.

**Methods:**

A549 cells were cultured in hypoxia condition, and then divided into two groups: hypoxia group and 3-MA plus hypoxia group. Electronic microscopy was used to detect autophagosome levels at different time points. The expression of LC3 was examined by Western blot. The proliferation activity of A549 cells after radiotherapy (0 Gy, 2 Gy, 4 Gy, 6 Gy, 8 Gy, and 10 Gy) was determined with an MTT assay.

**Results:**

Autophagosome formation and the LC3Ⅱ/LC3Ⅰ ratio increased under hypoxic conditions. After treatment with the autophagy inhibitor 3-MA, the quantity of autophagosomes and the LC3Ⅱ/LC3Ⅰ ratio decreased. The proliferative activity of the cells in the 3-MA plus hypoxia group was remarkably lower than that in the hypoxia group after treatment with radiotherapy.

**Conclusion:**

The level of protective autophagy of A549 cells increased in hypoxia condition; thus, inhibiting autophagy improves the radiosensitivity of A549 cells.

放射治疗是肺癌综合治疗的重要手段之一，如何减少放疗抵抗、增加放疗敏感性已成为国内外研究的热点^[1]^。低氧是肺癌等实体瘤微环境的基本特征，也是导致肺癌对放疗产生抵抗的原因之一，低氧可诱导肺癌细胞自噬的发生。近年研究^[2, 3]^表明，自噬可增加乳腺癌、恶性脑胶质瘤放疗抵抗。然而低氧环境中自噬是否影响肺癌的放疗治疗效应目前尚未完全明确。本研究通过观察γ射线对低氧环境中不同自噬水平A549细胞的杀伤作用，初步分析保护性自噬对A549细胞放疗敏感性的影响，为探讨自噬对非小细胞肺癌（non-small cell lung cancer, NSCLC）放疗敏感性的影响的分子机制奠定前期基础。

## 1 材料与方法

### 1.1 材料

RPMI-1640培养基、胎牛血清（Hyclone）；蛋白提取试剂盒（Pierce公司）；MTT粉（上海生物工程技术服务有限公司）；3-甲基腺嘌呤（3-methyladenine，3-MA，美国Sigma公司）；PCR仪（ABI公司）；三气培养箱（SANYO公司）；蛋白电泳仪、电转仪（Bio-Rad公司）；透射电子显微镜（日本JEM-2100）；直线加速器（西门子公司）。

### 1.2 细胞培养及实验分组

#### 1.2.1 细胞培养

人肺腺癌A549细胞（购于中科院上海细胞库），用含10%胎牛血清的RPMI-1640培养基于37 ℃、5%CO_2_/95%空气（常氧）饱和湿度培养箱常规传代培养。取生长状态良好的A549细胞经0.25%胰酶消化后，根据后续实验的不同，分别接种于60 mm培养皿、6孔板及96孔板中培养12 h。更换新鲜培养基后置于37 ℃、1%O_2_/5%CO_2_/94%N_2_三气培养箱中，继续后续实验。

#### 1.2.2 实验分组

① 低氧对照组；②低氧+3-MA（5 mmol/L）组。

### 1.3 电镜下观察自噬体的变化

取接种于60 mm培养皿中的A549细胞，加入3-MA（5 mmol/L）处理后继续低氧培养，分别于0 h，24 h，48 h用不含EDTA的胰酶消化细胞，PBS洗3次，加入新配的4%多聚甲醛，4 ℃过夜。然后将细胞沉淀放入1%的四氧化锇中，室温固定1 h，经一系列脱水及包埋后，固定于铜网上用电镜观察。

### 1.4 Western blot检测LC3蛋白的表达

取接种于6孔板中的A549细胞，3-MA（5 mmol/L）处理后低氧培养，于48 h收集低氧组及低氧+3-MA组的细胞，冷PBS洗2次，加入细胞裂解液于4 ℃裂解20 min，12, 000 r/min离心20 min，收集上清，提取细胞总蛋白，BCA法测蛋白浓度。取20 μg样品煮沸变形后进行Tricine-SDS-PAGE电泳，转膜，5%脱脂奶粉37 ℃封闭2 h，一抗4 ℃孵育过夜，洗膜，二抗37 ℃孵育1 h，ECL法显影，分析条带灰度值，以目的条带与β-actin调对灰度比值来表示蛋白相对表达量。

### 1.5 MTT法检测细胞增殖活性的变化

取接种于96孔板中的A549细胞，加入3-MA处理24 h后给予分别给予0 Gy、2 Gy、4 Gy、6 Gy、8 Gy、10 Gy射线照射，继续培养24 h。每孔加入MTT 20 μL，作用4 h，吸去培养液，加入二甲基亚砜（DMSO）150 μL，震荡10 min，于490 nm波长测定吸光度。计算细胞相对抑制率=（1-OD_实验组_/OD_对照组_）×100%，细胞相对存活率=1-相对抑制率。

### 1.6 统计学分析

采用SPSS 13.0统计软件进行分析，数据以Mean±SD表示，采用*t*检验分析组间差异，*P* < 0.05为差异有统计学意义。

## 2 结果

### 2.1 3-MA对低氧状态下A549细胞的自噬水平的影响

随着低氧培养时间延长（0 h、24 h、48 h），A549细胞中自噬体形成逐渐增加；给予自噬抑制剂3-MA后，A549细胞自噬体增殖明显减少（图 1），表明3-MA可抑制低氧状态下A549细胞的自噬活性。

**1:**
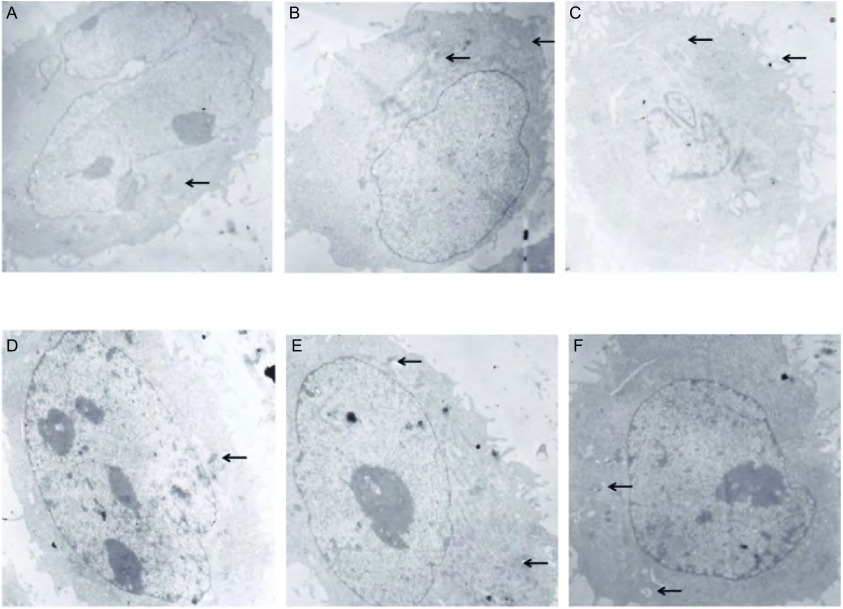
低氧状态下3-MA处理后A549细胞自噬体的变化（×6, 000） The change of autophagosome in A549 cells after treatment with 3-MA in hypoxia condition. A: hypoxia 0 h group; B: hypoxia 24 h group; C: hypoxia 48 h group; D: hypoxia plus 3-MA 0 h group; E: hypoxia plus 3-MA 24 h group; F: hypoxia plus 3-MA 48 h group.

### 2.2 3-MA对缺氧状态下A549细胞中自噬标记蛋白LC3蛋白表达的影响

LC3有两种存在形式（LC3Ⅰ、LC3Ⅱ），前体LC3合成后经加工形成LC3Ⅰ，后者与自噬体膜上的磷脂酰乙醇胺结合形成LC3Ⅱ。当自噬形成时LC3Ⅰ会减少而LC3Ⅱ会增加。经图像半定量分析灰度值（图 2），低氧组+3-MA组和低氧组LC3-Ⅰ/β-actin灰度比值分别为0.71±0.03和0.51±0.01，两组差异有统计学意义（*P* < 0.05）；LC3-Ⅱ/β-actin灰度比值分别为0.42±0.02和0.86±0.01，差异有统计学意义（*P* < 0.05）。结果显示，与低氧组相比，低氧+3-MA组LC3Ⅱ表达水平下调，LC3Ⅰ表达水平上调，LC3Ⅱ/LC3Ⅰ比值下降，表明3-MA可有效抑制缺氧状态下A549细胞的自噬标记蛋白的表达。

**2:**
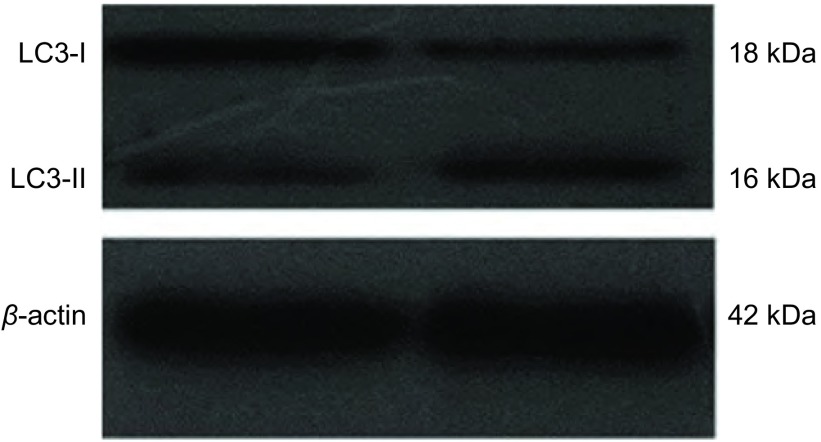
低氧48 h后两组A549细胞中自噬标记蛋白LC3的表达变化 The expression changes of LC3 protein in A549 cells after hypoxia for 48 h

### 2.3 低氧微环境下A549细胞自噬活性对放疗敏感性的影响

低氧状态下，放疗+3-MA组中A549细胞增殖活性较单纯放疗组下降（*P* < 0.05）（表 1），表明采用自噬抑制剂3-MA下调A549细胞自噬活性后，能增强A549细胞的放疗敏感性。

**1:** 低氧培养后不同放射剂量下自噬对A549细胞存活的影响 The effect of autophagy on the viability of A549 cell after treatment with different radiotherapy doses in hypoxia condition

Groups	Relative survival rate (%)					
	0 Gy	2 Gy	4 Gy	6 Gy	8 Gy	10 Gy
Hypoxia	100.00	76.88±3.52	60.85±3.28	35.20±2.76	10.57±2.55	5.11±1.02
Hypoxia+3-MA	91.10±3.82	60.33±2.55^*^	30.15±3.42^*^	20.44±2.88^*^	1.88±0.18^*^	0.11±0.05^*^
^*^P < 0.05 *vs* hypoxia group.						

## 3 讨论

自噬是细胞为适应营养或生长因子缺乏、低氧、内质网应激等有害刺激所发生的一种应激反应^[4]^。在氧供不足或营养缺乏时，肿瘤细胞可通过自噬缓解细胞的代谢压力，克服营养缺乏和低氧环境得以生存^[5]^；同时，自噬可选择性地清除某些细胞成分，如受损或多余的氧自由基、DNA、过氧化物酶体、内质网、线粒体等，减少异常蛋白、细胞器的堆积，以维持细胞自我稳态^[6]^。自噬作为一种防御机制，在应激状态下，特别是缺氧条件下对维持肿瘤细胞存活、逃避凋亡具有重要作用。

放射治疗是肿瘤最重要的治疗手段之一，然而却因肿瘤的放疗抵抗而易导致肿瘤的复发和残留。研究发现抑制肿瘤细胞的自噬水平可影响放疗敏感性。Ito等^[3]^证实，与单纯放疗组相比，放疗联合自噬抑制剂（3-MA和巴佛洛霉素A）可明显增加恶性脑胶质瘤U373-MG细胞DNA双链断裂损伤。Chaachouay等^[2]^则证实，与乳腺癌放疗敏感细胞株HBL-100相比，乳腺癌放疗抵抗细胞株MDA-MB-231自噬活性明显增强，采用自噬抑制剂（3-MA和氯喹）预处理MDA-MB-231细胞，可明显抑制其增殖活性，减少其放疗抵抗。自噬与肺癌亦关系密切。Kim等^[7]^使用自噬抑制剂如caspase-3抑制剂（Z-DEVD）和mTOR抑制剂（RAD001）能使NSCLC H460细胞增殖活性下降，凋亡增加，从而明显增强H460细胞的放射敏感性，同时在小鼠荷瘤模型中观察到，放疗分别联合Z-DEVD或RAD001，生存期较单纯放疗组分别延长3 d和5 d。

可见，自噬调节联合放疗有望成为肿瘤治疗的新策略。但目前有关肺癌与自噬关系的研究，绝大多数局限于在常氧状态下，而在低氧微环境中，自噬影响肺癌细胞放疗敏感性的研究目前仅见零星报道。

由于肺癌等实体肿瘤在发生发展过程中，细胞的快速生长、氧耗增加，加之血管新生紊乱，肿瘤内部常处于低氧状态。低氧可增加放疗抵抗而使肿瘤更具有侵袭性^[8]^，但其具体机制未明。研究表明低氧可诱发细胞自噬的发生。为了更好地模拟肿瘤生长的体内微环境，探讨自噬对低氧微环境中人肺腺癌A549细胞放疗敏感性的影响，课题组建立了低氧培养条件，采用肺腺癌A549细胞为研究对象，观察不同自噬活性下A549细胞对放疗射线的敏感性。

本研究通过观察自噬体的数量及检测LC3的表达、分析LC3Ⅱ/LC3Ⅰ比值变化来判断细胞的自噬活性。LC3蛋白是自噬体膜上标志性蛋白，是反映自噬活性较特异的指标。研究^[9]^报道，5 mmol/L 3-MA可抑制常氧环境中胃癌BGC-823细胞自噬水平，本研究证实给予3-MA（5 mmol/L）处理后，低氧环境中A549细胞自噬体数量减少，LC3Ⅱ/LC3Ⅰ比值下降，这表明3-MA（5 mmol/L）亦可成功抑制低氧环境中A549细胞自噬的发生。此外，本研究还发现，在不同剂量放疗照射同时给予3-MA处理A549细胞，细胞相对存活率较单纯放疗组均有不同程度的下降。表明采用3-MA抑制自噬可增强A549细胞放疗敏感性，但其具体分子机制尚不明确，分析可能与3-MA通过抑制bcl-2表达使肿瘤细胞凋亡增加以及3-MA通过抑制PI3K/AKT途径进而阻断COX2诱导的凋亡抵抗^[10]^有关，尚有待于进一步实验证实。

文献^[11]^报道，3-MA可影响肿瘤细胞增殖，且呈剂量依赖性。本实验未放疗组（0 Gy组）结果显示，3-MA（5 mmol/L）处理细胞后其相对存活率较对照组呈下降趋势，但无统计学差异，考虑本实验单一剂量3-MA并未完全反映其对A549细胞增殖的影响。本研究选用3-MA（5 mmol/L）在有效抑制细胞自噬的同时，其对细胞增殖影响甚微，表明3-MA对A549细胞的放疗增敏作用是通过抑制细胞自噬水平实现的。此外，不同浓度3-MA对A549细胞自噬及放疗敏感性是否有影响，尚有待进一步实验明确。本研究结果为后期探讨自噬对NSCLC放疗敏感性的作用的分子机制奠定了前期基础。

## References

[1] Spalding AC, Lawrence TS (2006). New and emerging radiosensitizers and radioprotectors. Cancer Invest.

[2] Chaachouay H, Ohneseit P, Toulany M (2011). Autophagy contributes to resistance of tumor cells to ionizing radiation. Radiother Oncol.

[3] Ito H, Daido S, Kanzawa T (2005). Radiation-induced autophagy is associated with LC3 and its inhibition sensitizes malignant glioma cells. Int J Oncol.

[4] Pan YZ, Wang X, Bai H (2011). Autophagy and cancer. Zhong Liu Fang Zhi Yan Jiu.

[5] Tsuchihara K, Fujii S, Esumi H (2009). Autophagy and cancer: dynamism of the metabolism of tumor cells and tissues. Cancer Lett.

[6] Hara T, Nakamura K, Matsui M (2006). Suppression of basal autophagy in neural cells causes neurodegenerative disease in mice. Nature.

[7] Kim KW, Hwang M, Moretti L (2008). Autophagy upregulation by inhibitors of caspase-3 and mTOR enhances radiotherapy in a mouse model of lung cancer. Autophagy.

[8] Dang YZ, Fei JX (2008). Hypoxic tumor cells and radiotherapy. Xian Dai Zhong Liu Yi Xue.

[9] Jin ZX, Lan RC, Wang F (2012). The effect and mechanism of autophagy inhibitor 3-MA to the effect and mechanism of gastric cancer BGC cell which inhibited by lactacystin. Zhongguo Lao Nian Xue Za Zhi.

[10] Choi S, Oh JY, Kim SJ (2011). Ginsenoside Rh2 induces Bcl‐2 family proteins‐mediated apoptosis *in vitro* and in xenografts *in vivo* models. J Cell Biochem.

[11] Hou H, Zhang Y, Huang Y (2012). Inhibitors of phosphatidylinositol 3' -kinases promote mitotic cell death in HeLa cells. PLoS One.

